# Comparison of manual versus automated thermal lid therapy with expression for meibomian gland dysfunction in patients with dry eye disease

**DOI:** 10.1038/s41598-024-72320-3

**Published:** 2024-09-27

**Authors:** Maria Laura Gomez, Jasmine Jung, Daisy D. Gonzales, Sarah Shacterman, Natalie Afshari, Lingyun Cheng

**Affiliations:** From UCSD School of Medicine Department of Ophthalmology, Shiley Eye Institute, La Jolla, CA USA

**Keywords:** Dry eyes, Meibomian gland dysfunction, Lipiflow, MiBoFlo, Thermoelectric gland expression, Eye diseases, Conjunctival diseases, Corneal diseases, Eyelid diseases

## Abstract

To compare two types of lipid expression procedures to treat dry eye disease. Standardized treatment and evaluation methods were used in patients treated with either manual thermoelectric lipid expression (MiBoFlo) or automated lipid expression (Lipiflow) of the Meibomian glands. This was a contemporaneous, non-randomized study of both treatment methods. Treatment was per the manufacturers’ recommendation. The primary outcome included two types of dry eye questionnaires as well as objective analysis of ocular surface including tear break up time, Schirmer testing, Osmolarity, and fluorescein staining. Baseline characteristics analyzed included floppy lid, conjunctivochalasis and lagophthalmos. Statistical analysis was performed correcting for baseline factors such as age and co existing pathology using multivariable analysis. Both treatments improved the results of the OSDI and SPEED dry eye questionnaire results. Both treatments resulted in improvement of many objective findings including SPK, lissamine green staining and tear break up time with the MiBoFlo showing more improvement than Lipiflow. OSDI was more sensitive to improvement of symptoms than the SPEED questionnaire. Manual expression with MiBoFlo device resulted in statistically more improvement in questionnaire scores than did automated expression with Lipiflow. Negative prognostic factors for symptomatic improvement included blepharitis, autoimmune disease and ocular allergies. Thermal lid therapy along with mechanical expression of lipids from the meibomian glands successfully treats dry eye symptoms and signs. Manual therapy with MiBoFlo resulted in more subjective and objective improvement scores than automated therapy with the Lipiflow device.

## Introduction

Dry eye disease is multifactorial but is characterized by tear film instability that causes symptoms of irritation, burning and foreign body sensation. It is characterized by loss of homeostasis of the tear film with accompanying ocular symptoms in which tear instability, hyperosmolarity, inflammation and ocular surface abnormalities along with neurosensory abnormalities playing important roles^1^. Tear supplements are often the first line of treatment but to be useful require indefinite frequent topical application and do not treat any of the underlying causes of the disorder^2^.

Anti-inflammatory and immunosuppressive treatments are useful in treating signs and symptoms and these are typically administered topically^3–6^ or in the form of a nasal stimulant spray^7,8^. Recently, therapy to restore a more functional lipid film layer by treating the meibomian gland dysfunction (MGD) has become an effective treatment modality. MGD is present in the majority of patients with dry eye disease^9^ and expressing the glands to reverse the clogging is often effective in treating patients^10–12^. All methods to do this involves thermal melting the inspissated lipids followed by some type of expression of the glands. Examples of such techniques are the LipiFlow, the iLUX MGD, TearCare, Intense pulsed light (IPL)^13–22^ and MiBoFlo^23^. One of the most commonly used modalities is called *Lipiflow* which is an automated instrument which warms the lids while massaging the meibomian glands in a pulsatile manner from the fornix towards the lid margin^14–16^.

We have previously shown that manual warming and expression of meibum is another effective method of treating meibomian gland dysfunction in dry eye disease. Using the MiBoFlo device, a probe is used to warm the lids while the doctor gently massages and expresses meibum. This is not an automated method so the physician can customize pressure and duration and treatment regions by monitoring the expression of the meibum at the lid margin. Our work showed that this manual method improves symptoms of dry eye comfort and function as evidenced by improved Ocular Surface Disease Index (OSDI and Standardized Patient Evaluation of Eye Dryness (SPEED) questionnaire scores and that this improvement was associated with reduced corneal fluorescein staining, reduced conjunctival lissamine green staining and improvement in tear break up time measurements^23^.

The purpose of the current study is to compare two consecutive case series of patients treated at the UCSD Shiley Eye Center Dry Eye Clinic using either *Lipiflow or* MiBoFlo instruments. These new treatments are different from traditional therapies with topical lubricants (2) or topical anti-inflammatory therapy (3–6) as well as being different from lid scrub treatment of blepharitis none of which physically evacuates the meibomian glands by liquifaction and subsequent meibum expression. Lipiflow may be considered the prototypical automated thermal and expressional treatment while MiBoFlo a typical manual and titratable therapy. All patients were treated according to a standard treatment protocol and underwent a standardized evaluation before and after treatment. This evaluation included complete ophthalmic examination, answering dry eye symptom questionnaires SPEED and OSDI, and evaluation of corneal staining, tear break up time, lissamine conjunctival staining and other measurements of ocular surface integrity before and six months after treatment.

## Methods

We retrospectively evaluated a consecutive case series of 409 eyes of 205 patients who came to our dry eye clinic and received either three monthly 10-min per eye thermoelectric warming therapy with MiBoFlo (219 eyes of 110 patients) or a single Lipiflow treatment (190 eyes of 95 patients) as recommended by the manufacturer and in the literature. Review of patient charts was approved by the UCSD Institutional Review Board. This study adheres to the tenets of the Declaration of Helsinki and informed consent was obtained from all subjects and/or their legal guardian(s). We had previously demonstrated that MiBoFlo is an effective treatment improving signs and symptoms of Dry Eye Disease in patients with MGD^23^ This is a retrospective study comparing our results with those obtained after a single Lipiflow procedure.

### Procedures

The MiBoFlo treatment and Lipiflow therapy was performed by a single treating physician using a standardized method. Therapy with the MiBoFlo was performed via a handheld probe, which warmed the upper and lower lids of each eye at the same time while keeping the temperature constant at 108 °F. According to the manufacturer, the thermoelectric probe that was used was regulated to deliver this preset consistent temperature, and if the temperature exceeded this, the instrument had an automatic shut off mechanism. Patients received three 10-min per eye thermoelectric warming treatments with MiBoFlo at a 2-week interval. Ultrasound gel was applied to the tip of the probe to permit heat through the lid to the meibomian glands. Gentle massage of the lids was performed with the probe alternating between circular and back and forth motion; the same was done for the other eye. The duration of each treatment was 10 min per eye. Inspissated lipid secretions were softened and mobilized through gentle ocular message. Liberated surfactants and improved lipid secretions create an enhanced tear film. Improved corneal wetting reduces or eliminates the signs and symptoms of ocular surface disease. No topical medications or anesthetics were required during or after the MiBoFlo treatment^23^.

The lipiflow patient cohort was treated with the lipifow system using a single use disposable activator placed on each eye that applied pressure in a pulsed vectored sequence while delivering heat to the eyelids to up to 108.5 °F. Prior to insertion of the disposable, one drop of topical anesthetic was applied to each eye. The disposable was inserted into the eye like a scleral contact lens or corneal surgical shield. Once the disposable was properly inserted, the 12‐minute treatment began. Both lids are treated simultaneously. During the treatment a programmed compression cadence occurs that includes cycles of constant, increasing, and alternating pressure, which results in the eyelids being heated and massaged for the entire 12‐minutes. As a result, the obstructed meibum was liquefied and expelled out of the gland orifices.

The two series of patients were treated nonconsecutively during the same time period by a single physician (MLG). The choice of treatment was left to patient preference as the number of treatments and in some cases cost was different between the two therapies. Review of patient charts was approved by the UCSD Institutional Review Board. Patients with acute allergic conjunctivitis, infection, or inflammation including infestation by demodex mites were excluded as they required different disease specific therapy. As part of a standardized evaluation and treatment algorithm, all patients received two validated dry eye questionnaires: the Ocular Surface Disease Index (OSDI)^24^ and the Standard Patient Evaluation of Eye Dryness (SPEED) questionnaire^25^ at the initial visit and at the 6-month visit. The SPEED questionnaire evaluated the frequency (0–3 scale) and severity (0–4 scale) of dry eye symptoms with the score calculated by adding the total complaint scores (0–28). The OSDI questionnaire assessed the frequency of dry eye symptoms (questions 1–5) and its effect on daily living tasks (questions 6–9) and certain environmental conditions that may trigger the symptoms (questions 10–12). Each question was graded on a 0–4 scale and a total score (0–100) was obtained based on the sum of scores × 25/number of questions answered.

All patients underwent a standardized history and ocular examination by a cornea trained ophthalmologist. History included general medical history, systemic or oral medications, other ocular conditions and surgeries, concurrent eye medications and allergies and daily screen usage time (> 6 h a day)^26^. Presence of any autoimmune disease such as Sjogren’s, rheumatoid arthritis and lupus, or other ocular conditions such as glaucoma, age related macular degeneration and rosacea as well as prior ocular surgeries (refractive, cataracts, lid surgery) known to exacerbate dry eye disease was recorded^27–34^. Age related macular degeneration (AMD) and glaucoma were noted as patients with visual dysfunction due to these conditions may have further reduction in image quality because of dry eye disease.

Objective evaluation included best corrected visual acuity, intraocular pressure, and tests for diagnosis and assessment of the ocular surface in dry eye (osmolarity, Schirmer’s I test, meibography, TBUT and corneal/conjunctival/lid margin staining)^35^. Osmolarity was measured with the TearLab System (TearLab Inc, San Diego, CA, USA), tear production was measured with 5-min Schirmer I test without anesthetic. Meibography was performed to objectively document lipid layer thickness, meibomian gland structure and blinking characteristics using the Lipiview (TearScience, Johnson & Johnson company, Morrisville, NC, USA)^35,36^.

A thorough slit-lamp examination of the ocular surface and adnexa followed, including corneal and conjunctival staining, evaluation of the lid margins and integrity of meibomian glands as well as osmolarity and TBUT. Presence of floppy lids, nocturnal lagophthalmos or conjunctivochalasis was carefully noted.

A standardized methodology is considered important^35^ so for TBUT, a fluorescein strip (BioGlo, HUB pharmaceuticals, Scottsdale, AZ, USA) was wetted with the application of one drop of non-preserved saline to the lower one-fourth of the strip. Excess fluid was gently removed such that the saturated tip delivered approximately 3 to 5 μL of liquid sodium fluorescein. With the subject gazing up, the examiner introduced the NaFl into the lower fornix. Subjects were instructed to blink naturally three times and then to cease blinking until instructed. Starting with the right eye, timing was stopped upon visualization of the first break (one or more black spots) appearing in the precorneal tear film or after 10 s had elapsed. TBUT was measured twice in each eye and averaged. The same procedure was repeated for the left eye.

Corneal fluorescein staining was evaluated on the slit lamp 90 s after instillation of fluorescein strip. The corneal staining (superficial punctate keratitis-SPK) was evaluated using the Van Bijsterveld scoring system (1: few separated spots, 2: many separated spots, 3: confluent spots)^37,38^.

Conjunctival staining was evaluated on the slit lamp 2 min after instillation of lissamine green standard strips (Green Glo, HUB pharmaceuticals, Scottsdale, AZ)^39^. Van Bijsterveld scoring was used measuring the presence of spots (1: few separated spots, 2: many separated spots, 3: confluent spots) in each conjunctival quadrant^38,40^.

### Statistical analysis

For continuous data such as ODSI or SPEED scores, tear film break-up time or tear osmolarity, values were expressed as mean and standard deviation while discrete data was expressed as a fraction or using count. To compare parameters between different time points on the same patient, paired tests were used.

For the comparison of two treatment methods, parameter changes between two time points were used as a response for a step-wise regression, using baseline parameters as regressors in a forward selection using significance level of 0.25 to enter. The final mixed model of regression was performed due to the fact that both eyes of the same patient were used in this study. All tests were two-tailed and *p* smaller than 0.05 was judged to be significant.

## Results

### Baseline characteristics of the patients

The mean age of the participants was 63.3 ± 15.72 years (range 18–91). Most of the participants were women (77.9%). Baseline characteristics of patients are shown on Table 1.

**Table 1:** Demographics and Baseline Characteristics of the Study Cohort.

Parameter	MiBoFlo		Lipiflow	
	n	%	n	%
No. of eyes/patients	219/110		190/95	
Age (Mean $\pm$ SD)	65.8 $\pm$ 14.7		60.4 $\pm$ 15.4	
*Sex*				
Female	169	77.17	148	77.90
Male	50	22.83	42	22.11
Other baseline characteristics				
AMD	31	14.16	12	6.52
Aqueous component	118	53.88	101	53.72
Auto-immune	60	27.40	32	17.98
Sjogren's disease	24	10.96	28	14.89
Blepharitis	167	76.26	168	88.42
Allergies	120	54.80	112	62.22
Conjunctivochalasis	118	53.88	110	57.90
Telangiectasia	100	45.66	140	73.68
Floppy lids	31	14.16	22	12.09
Glaucoma	18	8.22	16	8.51
Immunomodulators*	80	36.53	90	47/37
Hx Lid Sx**	48	21.92	50	26.32
Hx of lasik	18	8.22	41	22.04
Lagophthalmos	89	40.64	39	20.53
Partial blinkers	146	67.91	137	72.87
Rosacea	86	39.27	56	29.47
Sleep disorder	93	42.47	30	16.67

Hx: history of.

*Immunomodulators: Lifitegrast or cyclosporine.

**Lid Sx: previous eye lid surgery.

### Effect of two treatments judged by subjective dry eye scores

**Fig. 1:**
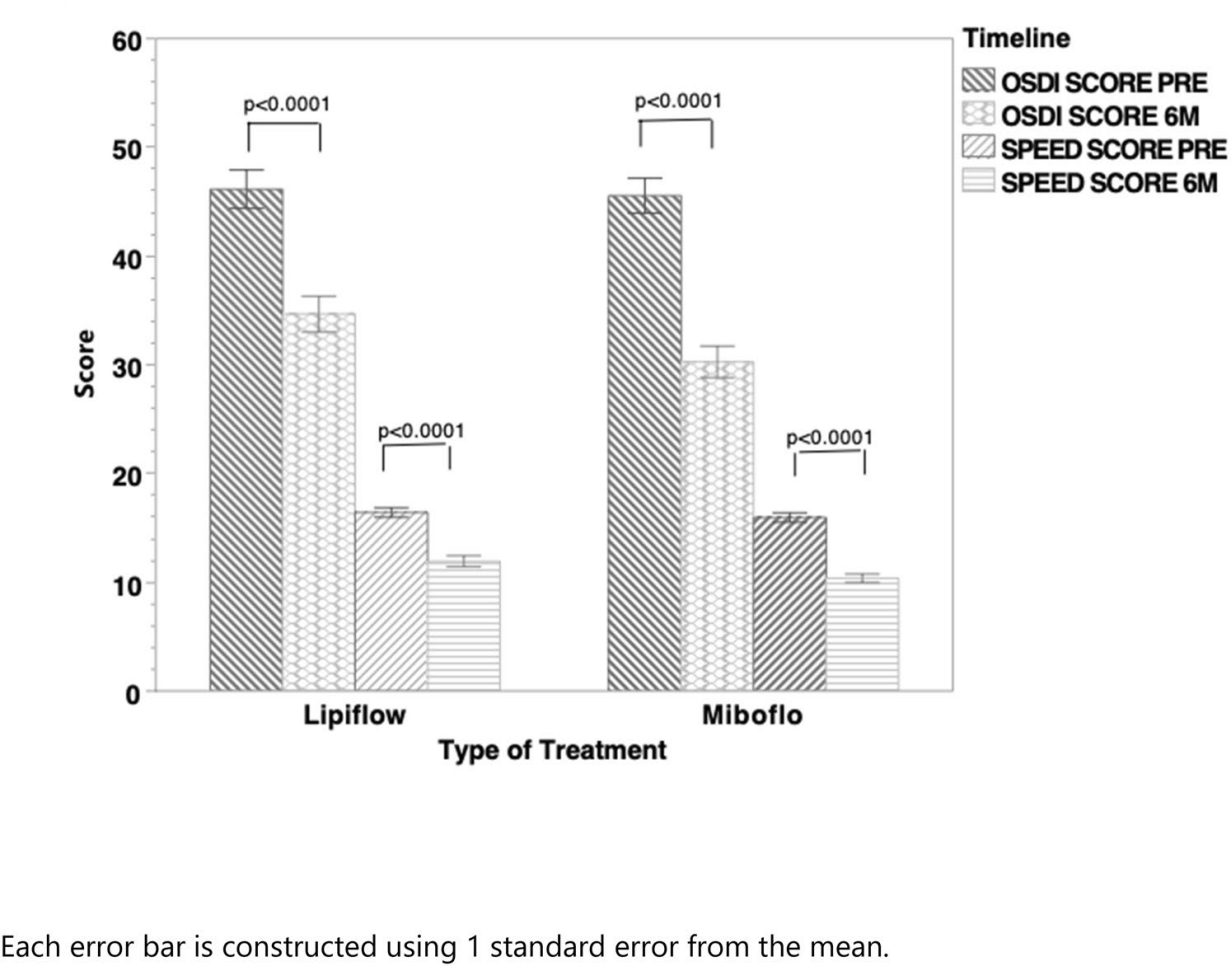
Bar graph indicates OSDI and SPEED score at baseline and at 6 months after the treatment, stratified by the treatment instrument. The magnitude of improvement of OSDI and SPEED from the baseline were also compared between the two treatment instruments as shown in Fig. 2. MiBoFlo group had a mean change (SPEED) in score of − 5.58 versus − 4.48 for the Lipiflow; and the difference between the two instruments was statistically significant *p* < 0.0001. In terms of the OSDI, Lipiflow improved by 11.48 points and MiBoFlo by 15.29; and the difference was significant (*p* < 0.0001). Each error bar is constructed using 1 standard error from the mean.

**Fig. 2:**
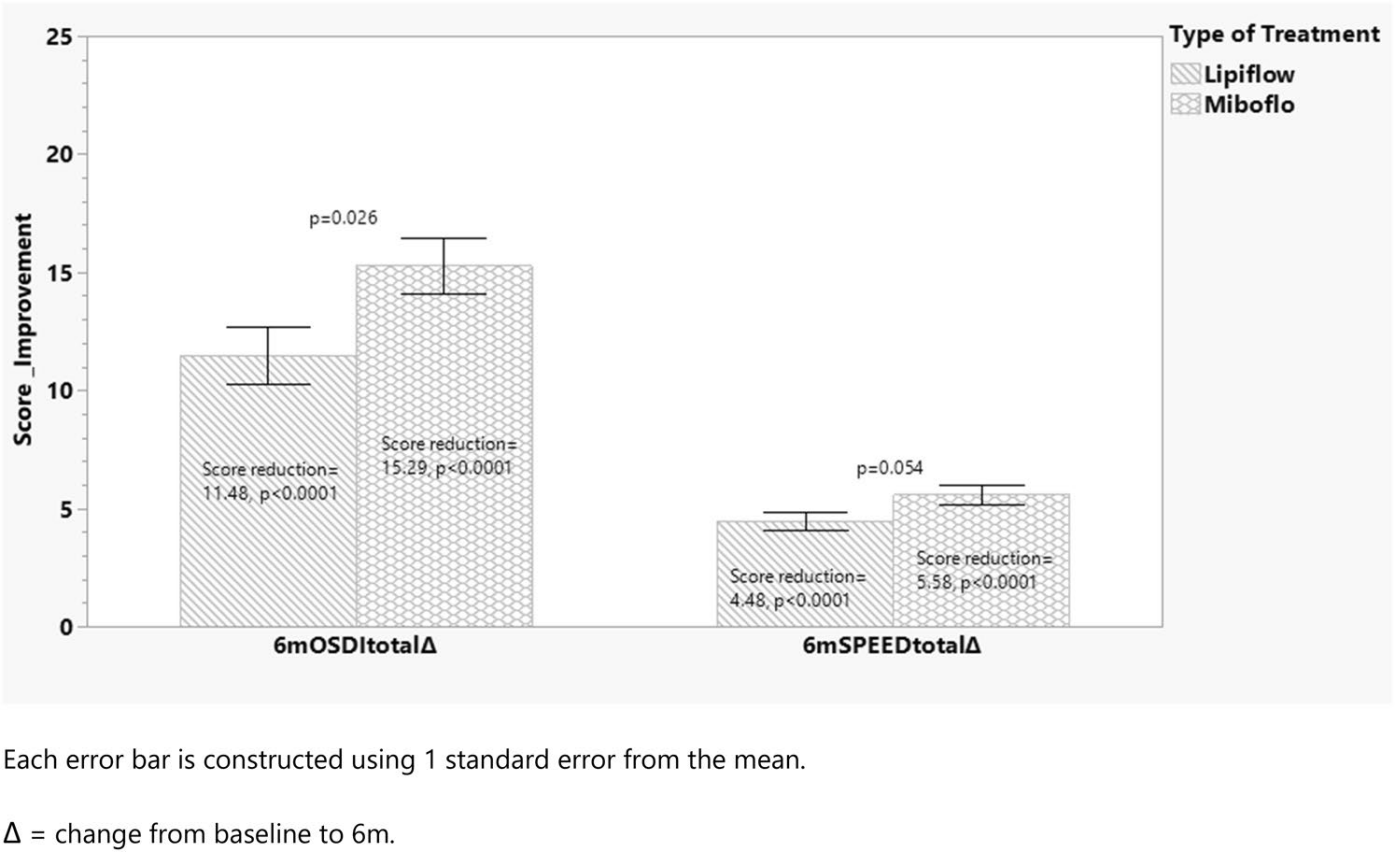
Bar graph showing OSDI and SPEED improvement magnitude stratified by the treatment instruments. For both symptom questionnaires MiBoFlo improvement is greater than the Lipiflow on unadjusted analysis. OSDI is more sensitive to treatment. Each error bar is constructed using 1 standard error from the mean. Δ = change from baseline to 6 m.

Figure 1 shows the results of dry eye symptom scores evaluated with two previously validated questionnaires, the SPEED and OSDI instruments. Both MiBoFlo and Lipiflow improved scores significantly from the baseline (Fig. 1, paired t-test). The score reduction in each questionnaire from baseline to 6 months is shown in Fig. 2. MiBoFlo treatment showed greater score reduction in both questionnaires compared to the Lipiflow treatment. (*p* = 0.001).

To cope with variation from two eyes of same subject and any potential small imbalance between the two treatment instruments, multivariate mixed model regression analysis of subjective dry eye score (SPEED and OSDI) were performed. For SPEED score evaluation, after stepwise regression analysis, MiBoFlo is significantly superior to Lipiflow (*p* < 0.0001) while adjusting for covariates which influence the baseline SPEED score, including blepharitis, conjunctivochalasis, and rosacea.

The presence of baseline blepharitis (β = − 0.81, *p* = 0.022), and conjunctivochalasis (β = − 0.93, < 0.0001), were negatively associated with SPEED improvement while rosacea (β = 0.78, *p* = 0.0082) was positively associated with SPEED improvement.

For OSDI score evaluation, after adjusting baseline OSDI score and the other significant baseline predictors such as blepharitis, autoimmune disease, and allergy, there was no significant difference in OSDI score improvement between two thermal treatments. Pre-existing conditions of blepharitis, autoimmune disease, and allergy were associated with less improvement of OSDI after thermal lid treatments.

Predictors of good vs poor symptom outcome:

1. Less improvement outcome on SPEED is predicted by conjunctivochalisis or blepharitis. More improvement is predicted by rosacea.
2. For OSDI Less improvement is seen in eyes within blepharitis, autoimmune and allergies. Rosacea is not a significant factor.
3. Changes of objective dry eye signs after the two treatments

Analysis of objective signs of dry eye between the two groups show that there was more improvement in SPK in the MiBoFlo group (− 1.06) than the Lipiflow group (− 0.67) *p* = 0.0069. Lissamine green staining showed more improvement in the MiBoFlo group (*p* = 0.001) with mean improvement in Lipiflow − 2.1 vs. −3.9 in MiBoFlo.

Similarly tear break up time was evaluated (TBUT) and there was improvement in both groups, but the MiBoFlo fared better (Table 2). TBUT Improvement after Lipiflow was 2.4 s longer and in MiBoFlo 3.4 s longer (*p* = 0.0001) (Table 2). Tear osmolarity improved (reduced) after both treatments, however, the difference was not statistically significant.

**Table 2:** Changes of objective dry eye signs following two treatments.

	Lipiflow	MiBoFlo	*p* Value
SPKΔ	− 0.67 ± 1.06	− 1.06 ± 1.78	0.0069
LGCSΔ	− 2.06 ± 3.24	− 3.92 ± 4.25	< 0.0001
Tbut Δ(s)	2.37	3.38	< 0.0001
OsmolarityΔ	− 4.62 + − 27.42	− 2.99 +− 23.96	0.5338

Δ: change from baseline to 6 m.

## Discussion

The primary purpose of this study was to compare two types of thermal lipid extraction techniques in terms of patient symptom improvement and objective anterior segment changes of dry eye. We compared the MiBoFlo to the Lipiflow. The MiBoFlo consists of warming and manual extraction of lipid in three sessions whereas the Lipiflow is a single session treatment using an automated instrument which warms and extracts lipids. The baseline characteristics of the two treatment populations were generally similar but our statistical technique took into account baseline factors as covariates in the analysis of outcomes. We evaluated symptoms using both the OSDI questionnaire and the SPEED questionnaire before treatment and six months after initiation of treatment. We found a statistically significant improvement with both treatments as measured by improvement in both the OSSDI and SPEED score between baseline and month 6. The magnitude of improvement between the two instruments was significant with more improvement in questionnaire scores after MiBoFlo than Lipiflow with approximately a difference of 4 points after MiBoFlo treatment and difference of one point on SPEED score. Statistical analysis was also performed account for take variables which may influence questionnaires. This confirmed the superiority of MiBoFlo to Lipiflow therapy in improving dry eye questionnaire scoring.

OSDI scores showed a more marked difference in questionnaire improvement which was more significant statistically and in magnitude. It is of interest to understand why the OSDI seems to show a larger difference (measures more improvement than SPEED) between the two treatment groups. The OSDI analyzes comfort as well as the impact of dry eye on function and quality of life so may be a more robust instrument to analyze the patient’s perception of success of treatment.

We also wished to determine which baseline ophthalmic characteristics predicted response to treatment. Interestingly, for the OSDI questionnaire, negative prognostic factors included blepharitis, autoimmune disease, and allergy. Thus, in treating patients, the physician should understand that all patients have improvement however eyes with the aforementioned characteristics may have less symptomatic benefit.

It is important to understand which objective anterior segment characteristics show improvement after treatment. There was a reduction of SPK, Lissamine Staining and an improvement in tear break up time after either treatment however the improvement was of larger magnitude with the MiBoFlo than the Lipiflow.

One remaining question is why the MiBoFlo which is a handheld device operated by the treating physician gave superior results to Lipiflow. The fact that the MiBoFlo showed more improvement in questionnaire efficacy as well as in anatomical efficacy suggests that the differences between the two types of treatment may be important. It is noteworthy that the Liipiflow did result in improvement of these signs but not to the extent of the MiBoFlo. We studied the manufacturers recommended treatment recommendation which was three monthly treatments with the MiBoFlo and one treatment with the Lipiflow. It is possible that multiple Lipiflow treatments would have resulted in more improvement, but the treatment is costly and the clinical trials demonstrating efficacy utilized one treatment session. Another possible explanation for higher efficacy of MiBoFlo is the fact that the treatment is customized to the patient. The treating physician may change pressure on the lids and modify other factors while being able to view the lipid extraction during treatment. With Lipiflow the treatment is automated and is the same for all patients. It should be noted however that Lipiflow requires insertion of an activator beneath the lids on the ocular surface. This is usually comfortable, and it is done under topical anesthesia. However, there is a risk of corneal abrasion or other mechanical problems if done improperly.

## Data Availability

Regarding the raw data, the data that support the findings of this study is available from the authors upon reasonable request and with permission of the IRB. Requests should be made to lagomez@health.ucsd.edu.
